# Modeling multi-mutation and drug resistance: analysis of some case studies

**DOI:** 10.1186/s12976-017-0052-y

**Published:** 2017-03-21

**Authors:** Mitra Shojania Feizabadi

**Affiliations:** Physics Department, Seton Hall University, 400 South Orange Ave, South Orange, NJ 07079 USA

**Keywords:** Cancer modeling, Conjoint cell growth, Chemotherapy, Drug resistance, Mutation

## Abstract

**Background:**

Drug-induced resistance is one the major obstacles that may lead to therapeutic failure during cancer treatment. Different genetic alterations occur when tumor cells divide. Among new generations of tumor cells, some may express intrinsic resistance to a specific chemotherapeutic agent. Also, some tumor cells may carry a gene that can develop resistance induced by the therapeutic drug. The methods by which the therapeutic approaches need to be revised in the occurrence of drug induced resistance is still being explored. Previously, we introduced a model that expresses only intrinsic drug resistance in a conjoint normal-tumor cell setting. The focus of this work is to expand our previously reported model to include terms that can express both intrinsic drug resistance and drug-induced resistance. Additionally, we assess the response of the cell population as a function of time under different treatment strategies and discuss the outcomes.

**Methods:**

The model introduced is expressed in the format of coupled differential equations which describe the growth pattern of the cells. The dynamic of the cell populations is simulated under different treatment cases. All computational simulations were executed using Mathematica v7.0.

**Results:**

The outcome of the simulations clearly demonstrates that while some therapeutic strategies can overcome or control the intrinsic drug resistance, they may not be effective, and are even to some extent damaging, if the administered drug creates resistance by itself.

**Conclusion:**

In the present study, the evolution of the cells in a conjoint setting, when the system expresses both intrinsic and induced resistance, is mathematically modeled. Followed by a set of computer simulations, the different growing patterns that can be created based on choices of therapy were examined. The model can still be improved by considering other factors including, but not limited to, the nature of the cancer growth, the level of toxicity that the body can tolerate, or the strength of the patient’s immune system.

## Background

The mechanism under which different types of cancers progress is complex. This progression depends on many factors including, but not limited to, the growth rate of cells, mutual interaction of cancer cells with surrounding normal cells, the way in which they are affected by immune system, and their response to anti-cancer treatment strategies. It also depends upon the mutations that may happen during cell division, leading into the inapplicability of chemotherapeutic treatments.

The most common therapeutic approach to reduce the population of cancer cells and control their progression is chemotherapy. However, on many occasions, the success of this treatment is barred as a result of ineffectiveness of the drug used, which is known as drug resistance [1]. Some of the factors that can contribute to the creation of drug resistance are related to the drug delivery defects, insufficient drug activation at the tumor site, or the resistance that results from genetic mutation of the tumor cells [2, 3]. Given the importance of implementing the best therapeutic strategy, detecting any pre-existing drug resistance, or any resistance induced by the drug during a course of chemotherapy, is important as it provides insight into the way in which the chemotherapeutic approach needs to be modified [4].

Cancer cells constantly divide into new generations of cancerous cells. Some of the newly born cells may contain mutated genes that express resistance to anticancer drugs, while the rest can still be susceptible to the therapy. This type of drug resistance is known as intrinsic resistance. A variety of approaches are being implemented to overcome the development of intrinsic drug resistance, including the uses of very high doses of chemotherapy, or utilizing combination therapies [5]. However, the success of chemotherapeutic treatments is less probable in cases when the implemented drug induces drug resistance. For example, some works show that in the case of drug induced resistance, it takes only few days for a tumor to regrow after the appearance of the resistance [6]. In some types of cancers, such as non-small cell lung cancer (NSCLC), a high percentage of patients develop drug resistance after long-term drug administration. In NSCLC, this kind of drug resistance is associated with a new set of mutations (T790M) created among wild cancerous tumor cells exposed to the specific chemotherapeutic agent. In such cancers, while the drug-responsive tumor cells shrink, the mutated tumor cells become resistant to the therapy; therefore, they grow and form a new tumor that no longer is responsive to the treatment [7].

An effective adjustment to a therapeutic approach is tied to a close monitoring of the evolution of untreated and treated normal and cancer cells, and the detection of any sign of resistance that may occur during the term of therapy. Modeling the evolution of the system has attracted more attention as it can provide insight into the progression of the diseases under a specific treatment; therefore, it is considered a parallel tool for tailoring the therapeutic approaches.

The various models expressed the growth of untreated normal and tumor cells with their possible interactions [8–12]. These models were then expanded to evaluate the dynamic of the system when different therapeutic approaches, including chemotherapy, viro-therapy, radiation, and immunotherapy, were utilized. Also, some models have mathematically and numerically examined different cases in which some level of drug resistance exists in the system [13–23]. The evolution of different types of cancer cells in a multi-resistance setting under chemotherapeutic treatment is not well considered in previous researches.

The aim of the present study is to construct a new model to improve biological reliability, and to enable the evaluation of the conditions in which the special chemotherapeutic drug can contribute to the treatment of cancer or cause more damage during the treatment by including drug resistance.

This paper focuses on simulating the evolution of different types of cancer cells in a multi-resistance setting under chemotherapeutic treatment.

## Methods

### Conjoint core model in an intrinsic chemo-resistance setting

In a conjoint setting, normal and tumor cells interact with one another during their growth. This mutual interaction between normal and tumor cells has been biologically detected and was initially modeled by Witten [8, 9]. In our previous work, we modified this core model to include the resistance that tumor cells may express against chemotherapeutic agents. To set this modification, we first considered that the control of normal cells over the growth of tumor cells is negligible, as it is large tumor cells that mainly express resistance to the treatment. We then included a second group of tumor cells in the core model. We assumed that this new group of tumor cells would be created during cell division and would carry a mutated gene that causes intrinsic resistance against a specific type of chemotherapeutic agent. Further, we assumed that this group of tumor cells would also grow under the logistic growth law. This model can be analyzed in the presence of a specific anti-cancer agent, where the population of drug-responsive tumor cells is reduced as a result of the interaction with the drug. The dynamic of the component of this system was characterized by the following set of equations:

1a $$ \frac{dT(t)}{dt}={r}_T T\left(1-\frac{T+{T}_R}{K_T}\right)-\tau T(t)-{a}_T\left(1-{e}^{- MC}\right) T,\kern0.5em ;\kern4.75em  T(0)={T}_0 $$

1b $$ \frac{d{T}_R(t)}{ d t}={r}_R{T}_R\left(1-\frac{T+{T}_R}{K_R}\right)+\tau T(t); \kern11.25em {T}_R(0)={T}_{R0} $$

1c $$ \frac{dN(t)}{dt}={r}_N N\left(1-\frac{N}{K_N}\right) + \kappa \left( T+{T}_R\right)\left(1-\frac{T+{T}_R}{T^{*}}\right)-{a}_N\left(1-{e}^{- MC}\right) N.\kern3em  N(0)={N}_0 $$

where N(t), T(t), T_R_(t) are respectively the total number of normal cells, drug-responsive tumor cells, and drug-resistant tumor cells with the unit of cells. Also, K_N_, K_T_, K_R_ are the carrying capacity of normal cells and two types of tumor cells with the unit of cells. The per capita growth rate for the drug-responsive tumor cells, drug-resistant tumor cells, and normal cells are expressed by r_T,_ r_R_, r_N_ with the unit of time^−1^. The T* is the critical size of the collection of tumor cells with the unit of cells. The second term in equation 1c represents the interaction between tumor and normal cells. In this term κ has the units of time^−1^. The drug-responsive tumor cells become intrinsically resistant tumor cells with a mutation rate of τ (time^−1^)*.* The last term in eq. 1a and 1c represents the interaction of normal and drug-responsive tumor cells with chemotherapeutic drugs. These cells die due to drug toxicity. The response function to the chemotherapeutic drug can be structured as a_i_(1-e^MC^), where M is associated to the drug pharmacokinetics and known as the drug efficiency coefficient with the unit of m^2^.mg^−1^, and C represents the amount of the drug at the tumor site (mg. m^−2^). The coefficient a_i_ when i = N, T with the unit of time^−1^ expresses the rate of chemotherapy-induced death [18, 24].

To achieve a more complete picture of the evolution of the cells in a drug resistance setting, the current model is modified below to include those types of drug resistance created as a result of interaction with chemotherapeutic agents.

## Conjoint core model and drug-induced resistance and simulations

### Extended model

A group of tumor cells with specific mutated genes may develop resistance to chemotherapeutic agents as they interact with the drug. To introduce this type of the drug resistance in our model, three groups of tumor cells were considered. As explained before, the first group are tumor cells that are responsive to the drug and grow under the logistic law, and their population decreases as they interact with the drug. The drug-sensitive tumor cells create a new generation of tumor cells as they divide. We assume that the newly born tumor cells can be placed in one of the following three groups. The first group includes those that are still responsive to the administered drug, and are known as wild tumor cells, T. The second group is those tumor cells that are still responsive to the drug, but carry a mutated gene that causes drug resistance as they interact with the introduced drug. These tumor cells are placed in the category of mutated tumor cells, T_M_. The third group of tumor cells is those that are not responsive to the drug and intrinsically resist the administered drug. This group is identified by T_R_. All of these tumor cells are assumed to grow under the logistic law. The term τ_1_T(t) in equations 1a and 1b expresses the transition of wild tumor cells to resistant tumor cells. The newly introduced term τ_2_T(t) in equations 2a and 2c represents the transition of wild tumor cells to mutated tumor cells. Also, the toxic effect of the administered drug, which leads to the reduction in populations of cells, has been expressed by a_T_(1-e^MC^)T on wild tumor cells as well [24]. The interaction of the drug with the mutated tumor cells partially kills them and partially turns them into drug-resistant tumor cells. The toxic effect of the drug which leads to the reduction of the population of mutated tumor cells has been expressed as a_TM_ (1-e^MC^)T_M_, where a_TM_ is the killing rate of mutated tumor cells induced by the first administered drug. Also, we considered that the mutated tumor cells also follow the logistic growth. Therefore, *K*_*M*_ and *r*_*M*_ are the carrying capacity of the mutated tumor cells and per capita growth rate of this group of tumor cells, respectively. The term that expresses the conversion of mutated tumor cells to drug-resistant in equations 2b and 2c has been expressed by τ_M → R_(1 − e^− MC^)T_M_. In this term τ_M → R_ with the unit of time^−1^ expresses the conversion rate of mutated tumor cells to resistant tumor cells due to interaction with the drug.

Furthermore, to evaluate cases that undergo combination therapy, a second chemotherapeutic drug can be added to the treatment, in the way that this second drug can be effective on drug-resistant tumor cells and can be mathematically introduced as ${\mathrm{a}}_{\mathrm{T}\mathrm{R}}\left(1-{\mathrm{e}}^{-{\mathrm{MC}}_2}\right){\mathrm{T}}_{\mathrm{R}}$ (equation 2b). In the following equations, a_T_, and *a*_*TM*_, are the death rate induced by the first administered chemotherapeutic drug, while a_TR_ is the death rate of the tumor resistance cells induced by this second drug. In addition, the concentration of the second drug is introduced by C_2_.

**Fig. 1:**
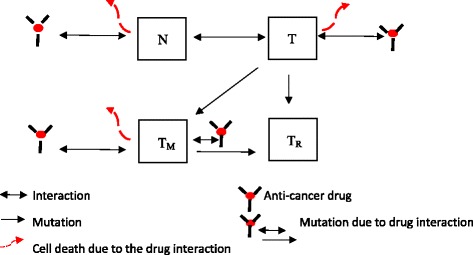
The schematic view of the system interactions. The system includes 4 types of cells: normal cells (N), wild tumor cells (T), mutated tumor cells (T_M_), and drug resistant tumor cells (T_R_). The population of normal, wild tumor and mutated tumor cells decreases as they interact with the drug. As the wild tumor cells divide, they can create mutated tumor cells or resistant tumor cells. As the mutated tumor cells interact with the drug, they can partially die and partially be transformed to resistant cells induced by the utilized anti-cancer drug

The schematic view of the system interactions is expressed in Fig. 1.

The dynamic of the system under these conditions can be expressed as below:

2a $$ \frac{dT(t)}{dt}={r}_T T\left(1-\frac{T+{T}_R+{T}_M}{K_T}\right)-{\tau}_1 T(t)-{\tau}_2 T(t)-{a}_T\left(1-{e}^{- MC}\right) T;\kern5.75em  T(0)={T}_0 $$

2b $$ \frac{d{T}_R(t)}{ d t}={r}_R{T}_R\left(1-\frac{T+{T}_R+{T}_M}{K_R}\right)+{\tau}_1 T(t)+{\tau}_{M\to R}\left(1-{e}^{- M C}\right){T}_M-{a}_{T R}\left(1-{e}^{- M{C}_2}\right){T}_R;\kern0.5em {T}_R(0)={T}_{R0} $$

2c $$ \frac{d{T}_M(t)}{ d t}={r}_M{T}_M\left(1-\frac{T+{T}_R+{T}_M}{K_M}\right)+{\tau}_2 T(t)-{a}_{T R}\left(1-{e}^{- MC}\right){T}_M-{\tau}_{M\to R}\left(1-{e}^{- MC}\right){T}_M;\kern0.75em {T}_M(0)={T}_{M0} $$

2d $$ \frac{dN(t)}{dt}={r}_N N\left(1-\frac{N}{K_N}\right)+\kappa \left( T+{T}_R+{T}_M\right)\left(1-\frac{T+{T}_R+{T}_M}{T^{*}}\right)-{a}_N\left(1-{e}^{- MC}\right) N.\kern0.75em  N(0)={N}_0 $$

### Numerical simulations and choice of parameters

To evaluate the dynamic of the cells, different cases and therapeutic approaches have been analyzed through a series of numerical simulations and under various treatment approaches. Table 1 expresses the values of parameters in each of the treatment strategies that has been numerically evaluated below.

**Table 1:** Parameters used in simulations in different therapeutic cases introduced above

Resistance Detected	Growth Parameters	Specifications of Drug I	*τ*_2_	*τ*_*M* → *R*_	*τ*_1_	Specifications of Drug II
Drug-Induced	K_T_ = K_N_ = K_R_ = 10^6^ (cells) r_T_ = r_r_ = r_M_ = 0.25 r_n_ = 0.5 day^−1^ κ = 0124 day^−1^ T^*^ = 3*10^5^ cells	Constant Drug C = 0.2 (mg.m-^2^) a _T_ = 0.15 (day^−1^) t(start) = 50 days	10^−3^/day Mutation starts at t = 0 day	10^−4^/day Conversion starts at t = 50 days	0 (No intrinsic resistance)	0 (No combination therapy)
Drug-Induced	Same as above	Constant Drug C = 0.2 (mg.m-^2^) a_T_ = 0.15 (day^−1^) t(start) = 50 days	10^−3^/day Mutation starts at t = 0 day	10^−4^/day Conversion starts at t = 150 days	0 (No intrinsic resistance)	0 (No combination therapy)
Drug-Induced	Same as above	Decaying Drug C = 0.2exp(-0.001 t) (mg.m-^2^) a_T_ = 0.15 (day^−1^) t(start) = 50 days	10^−3^/day Mutation starts at t = 0 day	10^−4^/day Conversion starts at t = 50 days	0 (No intrinsic resistance)	0 (No combination therapy)
Drug-Induced and Intrinsic	Same as above	Decaying Drug C = 0.2exp(-0.001 t) (mg.m-^2^) a_T_ = 0.15 (day^−1^) t(start) = 50 days	10^−3^/day Mutation starts at t = 0 day	10^−4^/day Conversion starts at t = 50 days	10^−4^/day	C_2_ = 0.6 (mg.m-^2^) a_TR_ = 0.15(day-1) t(start) = 50 days

The description of parameters are: *τ*_2_: Mutation rate of wild tumor cells to tumor cells with the gene that can potentially go through second mutation to drug-resistant cells as they interact with the drug. *τ*_*M* → *R*_: Mutation/ conversion rate of mutated tumor cells with targeted gene to drug-resistant tumor cells. *τ*_1_: Mutation rate of wild tumor cells to drug-resistant tumor cells. Specifications of Drug I (Effective on wild and mutated tumor cells). Specifications of Drug II (Effective on drug resistant tumor cells). Also, the common parameters related to the tumor growth are: K_N_, K_T_, K _R_: the carrying capacity of normal cells and two types of tumor cells. The per capita growth rate for the drug-responsive tumor cells, two groups of drug-resistant tumor cells, and normal cells are expressed by r_T,_ r_R,_ r_M_, r_N_. The T* is the critical size of the collection of tumor cells. κ is the parameter expresses interaction between tumor and normal cells

The set of equations introduced above (2a-2d) shows the population dynamic of four variables (T, T_R_, T_M_, N). The terms of these equations and associated parameters describe the growth of each introduced population of the cells, or the way that they are affected as a result of a) the existing dependency among populations, b) transition among populations, and c) the interaction with the drug.

The values of the parameters associated to the growth of tumor cells are tumor-specific. Also, previously reported measurements show that the obtained values from participants in clinical trials may also be different from those obtained from in vivo experiments [24]. The current work has no concentration on a specific type of tumor. However, the values of the parameters we have chosen are in the range with those reported by other studies. For parameters with no specific reported values, ad hoc values have been chosen. The goal is to analyze the behavior of the system under these specifically chosen cases. Below, the choice of parameters has been explained with further details.

Two parameters that describe the growth are the growth rate and the carrying capacity. Other studies have reported a value of less than one for the growth rate, and a carrying capacity between 10^5^ cells and 10^9^ cells [15, 25]. However, it should be noted that the value of the carrying capacity is organs-specific [26]. To be in this range, the adapted values in this study are K_M_ = K_T_ = K_N_ = K_r_ = 10^6^ (cells), r_T_ = r_R_ = r_M_ =0.25 (day^−1^), r_N_ = 0.5 (day^−1^). To choose the value of T*, we referred to the reported study of Demicheli *et al.* [27], which explains that more information is available on the initial and last stages of the tumor growth than on tumor growth in the intermediate phase. The study explains that the growth pattern in the intermediate phase is very complex and tumor specific. The critical tumor cell size of human colon carcinoma cell line "LoVo," where the pattern of growth deviated from the Gomertizian, was measured to be in the intermediate phase of growth when the tumor cell population was approximately 10^3^ cells [27]. Relying on the findings of this study, and since the carrying capacity (the last phase of growth) in the current work is set to be 10^6^ cell, T* has been chosen to be 10^5^ cells, to be placed in the intermediate growth phase.

The parameters related to the chemotherapeutic agents are: the death rate of the tumor cells induced by the drug, the drug concentration and, accordingly, toxicity coefficient, and the drug pharmacokinetics parameters. The induced death rate for drug sensitive tumor cells a_TR_ and *a*_*T*_ (0.15 day^−1^) are considered to be equal, and the order of the value of these parameters is consistent with those reported by previous studies [15]. Also, the two parameters are associated to the chemotherapeutic agent: "M", the pharmacokinetics parameters with a value of 1, and C, the drug concentration or toxicity coefficient [24]. We first evaluated the cases when the drug concentration remains constant (C = 0.2 mg.m^−2^) in the tumor site. Then, the dynamic of the cell populations was evaluated in cases in which the amount of the chemotherapeutic agent went under decay in the tumor site. This decay was considered to be exponential with the drug decaying rate of 10^−3^ day^−1^. The value for the decaying parameter is an ad hoc value and is case based.

The value of the parameters related to the transition of the cells from one sub-population to another are also ad hoc values. These transitions include those from wild tumor cells to resistant tumor cells; wild tumor cells to mutated tumor cells that can be converted to resistant tumor cells as they interact with the drug; and mutated tumor cells to resistant tumor cells. The method of measurement of the rates of these mutations and transitions is being explored. Therefore, the evaluation of the dynamic of the system is limited to the case study with some ad hoc values. The selected values for these parameters are reflected in Table 1.

Under this general method of choosing parameters, different values are considered for the parameters that are linked to the therapeutic approaches. The outcomes of simulations under these choices are discussed below.

## Results and Discussion

### Numerical simulations under different therapeutic approaches

#### Constant drug and drug-induced resistance

**Fig. 2:**
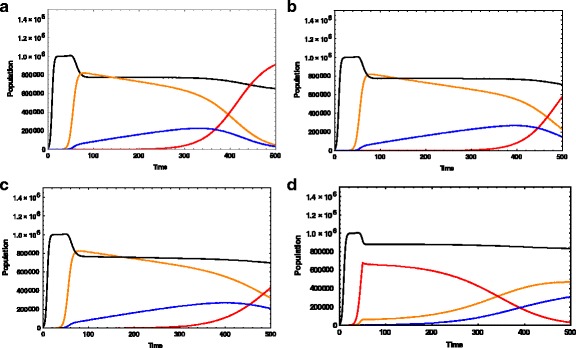
Dynamic of Cells under Different Therapeutic Strategies. This figure expresses the evaluation patterns of normal cells (black line), Wild tumor cells that are responsive to the drug (orange line), Mutated tumor cells which can potentially convert to drug-resistant tumor cells while responsive to the drug (blue line), and drug-resistant tumor cells (red line). **a**: Under the first therapeutic approach, it is assumed that the drug is administered at t = 50 days. The amount of the drug and the killing rates are considered to be constant. It is also considered that as the drug is introduced, the mutation to the second group of tumor cells, T_M_, starts immediately at the rate of 10^−3^ per day. Distinct from the **a**, in the simulation **b**, it is assumed that the transformation of the mutated tumor cells to resistant cells starts at the later time of t = 150 days. In **c**, the therapy with anti-cancer drug starts at t = 50 days. However, the amount of the drug will not stay constant and decreases exponentially over time, with a decay factor of 10^−3^ day^−1^. The simulation **d** evaluates the case when the system expresses an intrinsic drug resistance, in addition to an induced drug resistance with the mutation rate of wild tumor cells to drug-resistant tumor cells of 10^−4^ per day

Under the first therapeutic approach, it is assumed that the drug is administered at t = 50 days. The amount of the drug and the killing rates are considered to be constant. It is also considered that as the drug is introduced, the mutation to the second group of tumor cells, T_M_, starts immediately at the rate of 10^−3^ per day. Part of the population of the mutated tumor cells will then be transformed into resistant tumor cells. However, as they are still responsive to the drug, another part dies due to the toxic effect of the drug. Those that remain will grow and create new cells that carry the mutated gene, which potentially can create drug-induced resistance. The outcome of the simulation expressed in Fig. 2a shows that the population of mutated tumor cells and wild tumor cells have been successfully controlled in the time frame of simulation (t = 500 days). They both die out of the system. The drug-resistant tumor cells are initiated and grow as well.

In our work, a Gompertzian model is considered for the growth of cells with the chosen value for the carrying capacity equal to10^6^ cells. Therefore, the critical point of the growth, when the cells enter a slower phase of growth, is around 2*10^5^ cells. This critical point is considered as a point when tumor population becomes clinically detectable [28]. In such a case, around t = 375 days, their populations can be detectable. However, they immediately grow and become the dominant population at the end of the simulation time, t = 500 days. Figure 2b evaluates the clinically observed cases when the drug-induced resistant tumor cells mutate as the wild tumor cells interact with the administered drug for a long time. Distinct from Fig. 2a, in this simulation, it is assumed that the transformation of the mutated tumor cells to resistant cells starts at the later time of t = 150 days. The shrinkage of drug-responsive tumor cells can be seen. In this case, the population of drug-resistant tumor cells is not yet dominant at the end of the simulation.

#### Decaying drug and drug-induced resistance

In Fig. 2c, the therapy with anti-cancer drug starts at t = 50 days. However, the amount of the drug will not stay constant and decreases exponentially over time, with a decay factor of 10^−3^ day^−1^. At t = 450 days, the drug-resistant tumor cells become detectable, and at the end of the simulation time, they are not dominant tumor cells. The population of drug-responsive tumor cells is still in the declining phase as they interact with the drug.

In the current case, as the simulation shows, some wild and mutated tumor cells still exist in the system. As they are responsive to the drug, the treatment can be continued. The drug-resistant tumor cells were created with a delay, as compared with the previous case when the amount of the drug was constant, and the growth of drug-resistant tumor cells occurred earlier.

It should be noted that in some cases, detecting drug resistance can be an advantage, as the therapeutic approach can be altered accordingly. By switching to another drug or by utilizing a combination therapy, there can be a path to a more successful treatment. This, certainly, is a therapeutic choice, depending on many health factors of the given patient.

#### Intrinsic and drug-induced resistance

The simulation 2 d evaluates the case when the system expresses an intrinsic drug resistance, in addition to an induced drug resistance.

One of the therapeutic approaches to controlling intrinsic drug resistance is the use of a higher dosage of a drug in early stages of the cancer progression. Under the simulation conditions, it is assumed that two types of drugs are implemented, one with a higher dosage that is toxic to drug-resistant cells. The amount of this drug is considered to stay constant over time. The second drug is toxic to tumor-responsive cells. The amount of this drug decays exponentially over time. The result of this simulation shows that this approach is effective in overcoming drug-resistant tumor cells. By the end of the simulation time, they die out of the system and the remaining tumor cells are responsive to the drug. In such a case, tailoring the therapy to achieve the best outcome is again a factor of concern. As there are still some drug-responsive tumor cells in the system, the treatment with the second drug can be continued. However, since the drug-resistant tumor cells have died out of the system, the treatment with the first drug can be terminated. The continuation of the treatment with the second drug, which is effective on drug responsive cells, raises the possibility of the creation of another drug-resistant tumor cell. It is possible that periodic treatments could be a more successful therapeutic approach to control both the progression of cancer as well as the existing resistance.

## Conclusions

Cancer drug resistance, which is an obstacle to successful treatment outcomes, is not limited to intrinsic resistance. The success of the treatment becomes more unpredictable if the introduced drug induced some resistance. The utilization of different chemotherapeutic drugs, combination therapy, and periodic therapy are some protocols that are currently implemented. The use of modeling and computer simulations enhance our understanding of the evolution patterns that may occur during treatments. In the present study, the evolution of the cells in a conjoint setting, when the system expresses both intrinsic and induced resistance, is mathematically modeled. Followed by a set of computer simulations, the different growing patterns that can be created based on choices of therapy were examined. The model can still be improved by considering the nature of the cancer growth: for example, it would be more realistic to include such things as the blood supply, the three dimensionality of the growth, and other important biological variables.

Some open concerns include whether the mutations happen at a constant rate or if the rate can be affected by the drug and other surrounding conditions. Furthermore, in all of these cases, the level of toxicity that the body can tolerate, along with other factors such the strength of the patient’s immune system, play important roles in making decisions with regards to impactful therapy.

## Abbreviations

NSCLC: Non-small cell lung cancer
